# Home-based screening tools for amblyopia: a systematic review

**DOI:** 10.1038/s41433-023-02412-3

**Published:** 2023-02-24

**Authors:** Samantha Siaw Zhen Sii, Chung Shen Chean, Helen Kuht, Catey Bunce, Mervyn G. Thomas, Sohaib R. Rufai

**Affiliations:** 1 Department of Ophthalmology, University Hospitals of Derby and Burton, Derby, UK; 2 University of Leicester Ulverscroft Eye Unit, Leicester Royal Infirmary, Leicester, UK; 3 The Royal Marsden NHS Foundation Trust, London, UK; 4 Clinical and Academic Department of Ophthalmology, Great Ormond Street Hospital for Children, London, UK

**Keywords:** Medical research, Eye diseases

## Abstract

Amblyopia is an important public health concern. While home-based screening may present an effective solution, this has not been rigorously assessed in a systematic review. A systematic review was performed using Ovid MEDLINE, PubMed, The Cochrane Library, Embase, Web of Science Core Collection, and Clinicaltrials.gov. All studies reporting the diagnostic accuracy of home-based screening tools for amblyopia among children were included. Studies involving orthoptist or ophthalmologist-led screening and adult subjects were excluded. The main outcome measure was the diagnostic accuracy expressed as sensitivity and specificity. Among 3670 studies identified, 28 were eligible for inclusion in our systematic review. The age range of patients were less than 1 month to 16 years old. 7 studies used internet-based tools, 16 used smartphone/tablet applications, 3 used digital cameras, and 3 used home-based questionnaires and visual acuity tools. All studies included a reference standard except one, which was a longitudinal study. 21 studies had full ophthalmological examination whilst 6 studies had validated visual acuity measurement tools as gold standards. Of the 27 studies which compared against a reference test, only 25 studies reported sensitivity and specificity values. Using the QUADAS-2 tool, 50% of studies were deemed to have applicability concern due to patient selection from tertiary centres and unclear methods for recruitment. There is a need to improve the quality of diagnostic accuracy studies, standardise thresholds for detecting amblyopia, and ensure consistent reporting of results. Further research is needed to evaluate the suitability of these tools for amblyopia screening.

## Abstract (zh)

弱视是一个重要的公共健康问题。虽然基于家庭的筛查可能是一个有效的解决方案, 但这还没有在系统性回顾中得到严格的评估。我们使用Ovid MEDLINE、Pubmed、The Cochrane Library、Embase、Web of Science Core Collection和Clinicaltrials.gov进行了系统综述。并纳入所有研究儿童弱视评估以家庭为基础的筛查方法诊断准确性的文章。涉及视力矫正医师或眼科医生主导的筛查和以成人受试者为对象的研究被排除在外。主要结果指标是敏感性和特异性表示的诊断准确性。在确定的3670项研究中, 有28项纳入系统回顾。患者的年龄范围在1个月至16岁之间。其中7项研究使用基于互联网的评估工具, 16项研究使用智能手机/平板电脑应用程序, 3项研究使用数码相机, 3项研究使用基于家庭的调查问卷和视力工具。除了一项纵向研究以外, 所有的研究均包含参考标准。21项研究进行了全面的眼科检查, 而6项研究将有效的视力测量工具作为黄金标准。在与参考测试进行比较的27项研究中, 只有25项研究报告了敏感度和特异性值。经QUADAS-2工具评估, 50%的研究被认为有适用性问题, 原因是病人来自三级中心和招募方法不明确。需要提高诊断准确性研究的质量, 对检测弱视的阈值进行标准化, 并确保结果报告的一致性。未来需要进一步的研究评估这些方法是否适合用于弱视筛查。

## Introduction

Amblyopia, or ‘lazy eye’, is associated with a lack of visual stimulation in the early years of life, resulting in cortical visual impairment [1]. This could be due to amblyogenic risk factors including uncorrected refractive error, astigmatism, media opacities, ptosis or other congenital pathologies that cause stimulus deprivation, and abnormal binocular interaction from strabismus [2–4]. This condition represents a significant public health concern, with population prevalence estimated between 2–5% [5–7].

Even though amblyopia is initially largely asymptomatic, untreated amblyopia resulting in vision loss could lead to problems at school, bullying, reduced quality of life, lifelong consequences on future occupation choices, and mental health issues [8, 9]. Contrary to the traditional notion that amblyopia treatment may be ineffective for children above 7 years old [10], the Paediatric Eye Disease Investigator Group (PEDIG) studies demonstrated that treatment of amblyopia may still be effective in children aged 7 to 17 years [11, 12], with the effectiveness of treatment becoming significantly reduced with time [13]. Hence, it is better to detect amblyopia early via screening.

Traditionally, vision screening for amblyopia was performed in the healthcare setting by experienced or trained healthcare professionals, including orthoptists, optometrists, and ophthalmologists, or by non-trained professionals in schools. Many programmes have not been successful due to inconsistencies in screening modalities utilised and lack of systematic assessment of their impact [14]. Overcoming barriers to these traditional amblyopia screening methods such as cost, limited access to healthcare and a limited number of qualified screeners is an ongoing issue [15]. With the COVID-19 pandemic and its related restrictions and lockdowns, traditional vision screening has become more difficult and the use of home-based screening tools for amblyopia are increasingly advocated, so that children do not miss out on opportunities for amblyopia screening [16, 17]. However, the role of home-based screening tools for amblyopia has not yet been rigorously assessed by a systematic review.

Here, we performed a systematic review to evaluate the accuracy and reliability of home-based amblyopia screening tools compared with the existing gold standard.

## Methods

This systematic review was conducted in accordance with Preferred Reporting Items for Systematic Reviews (PRISMA) guidelines [18]. The study was registered on PROSPERO (CRD42021233511) and the protocol is published in *BMJ Open* [19].

### Eligibility criteria for studies in this review

Eligibility criteria were established prior to the conduct of this systematic review. All studies reporting diagnostic accuracies of home-based amblyopia screening tools among subjects less than 18 years old were included. Home-based screening tools included web or internet-based screening tools, mobile applications and other low-cost instruments such as digital cameras which could be used from home. All studies evaluating orthoptist or ophthalmologist-led screening, commercial photoscreeners, instruments incorporating artificial intelligence, autorefractors, and of adult subjects were excluded. Only Oxford Centre of Evidence Based Medicine (CEBM) level 4 evidence and above were included [20]. This includes case-series, cross-sectional studies, case-control studies, cohort studies, randomised controlled trials, and systematic reviews. All studies which did not report outcomes pertinent to the diagnostic accuracy of home-based amblyopia screening tools, such as studies reporting only validity or repeatability, epidemiological studies, case reports, expert reviews, opinion pieces, and conference abstracts without full publications, were excluded.

### Information sources and search strategy

Electronic searches were performed through Ovid MEDLINE (1946 to present), PubMed, The Cochrane Library, Embase (1974 to present), Web of Science Core Collection (1970 to present), and Clinicaltrials.gov. Searches were conducted from inception until 31st August 2021. References of relevant studies were also searched and included if they met the inclusion criteria.

Medical subject heading (MeSH) terms such as ‘amblyopia’, ‘visual acuity’, ‘vision screening’ and terms to capture home-based screening tools such as ‘home’, ‘web’, ‘internet’ ‘app’, ‘smartphone’, and ‘mobile’ were used for the search where applicable. The full search strategy can be obtained via Online Supplementary Appendix 1. EndNote V.X9 (Thomson Reuters, New York, New York, USA) was used for data management. No date or language restrictions were stipulated.

### Selection process

All studies went through a three-stage screening process involving title, abstract and full texts by two independent reviewers (SS, CS) according to the screening criteria available via Online Supplementary Appendix 2. Any disagreements were resolved with a third arbitrator (HJK). If there were any ambiguity on the screening tool, an email was sent to the first author of the paper to ask for more clarification before these studies were included. If there were missing data on specificity or sensitivity values, an email was also sent to the first author of the paper to acquire them. A reminder email was sent again if they did not respond after two weeks. If there was still no response after four weeks in total, those data were excluded from our analysis.

### Data Collection

The main outcome measure reported was the diagnostic accuracy of home-based screening tools for amblyopia detection, expressed as sensitivity and specificity values.

Data was extracted from eligible studies using a tool adapted from the Cochrane Collaboration in the form of a table (Online Supplemental Appendix 3). Data collected included study design, number of included patients, duration of study, method of intervention used, index test, and reference standard where applicable. Data pertinent to the quality of diagnostic studies including investigators conducting test, subjects receiving test, method of interpretation of test, blinding of participants or investigators, and withdrawal rate of participants were also collected.

### Risk of bias assessment

Risk of bias assessment was assessed using the QUADAS-2 tool for diagnostic accuracy studies (available via Online Supplementary Appendix 4) [21]. These judgments were made independently by two review authors (SS, CSC) and any disagreements resolved by the third arbitrator (HJK). Risk of bias and applicability concern were graded as low, unclear, or high.

Risk of bias and applicability concerns were graded for the following domains:

i. Patient selection
ii. Index test
iii. Reference test
iv. Flow and Timing

### Summary measures

In addition to specificity and sensitivity values, confusion matrices (tables containing true positive, false positive, true negative, and false negative outcomes) were extracted from the included studies. Authors of papers that did not publish these values were contacted via email and given two weeks to respond. A reminder email was sent again if they did not respond after two weeks. If there was still no response after four weeks in total, those data were excluded from our analysis. If there were multiple thresholds used for amblyopia detection within the same study, or more than one sensitivity or specificity value were reported, the results will be reported based on the thresholds specified.

### Patient and public involvement

There was no patient and public involvement during the conduct of this systematic review.

## Results

### Descriptive synthesis

**Fig. 1:**
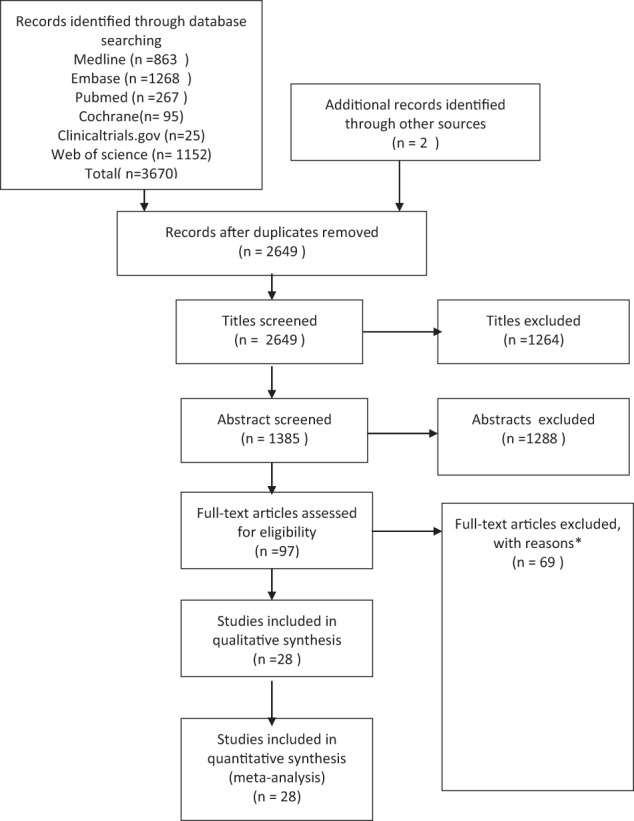
PRISMA study inclusion flow diagram. *Reasons for exclusion were as follows: Wrong intervention (*n* = 28); conference abstracts, short article or pre-prints (*n* = 19); Tool does not qualify as home-based tool (*n* = 12); wrong population (*n* = 4); wrong outcomes (*n* = 4); no results available (*n* = 2). Online Supplementary Table 1 contains the full list of excluded studies with reasons.

The search was executed on 14 August 2021 and the screening was completed on 31 November 2021. Our search returned 3670 studies in total, of which 1021 were duplicates. Following title, abstract and full text screening, 28 studies were included in our systematic review (Fig. 1). The full list of excluded articles and reasons for exclusion is available on Online Supplementary Table 1. Among the home-based screening tools for amblyopia, there were seven internet-based tools measuring visual acuity [22–28], 16 mobile phone or iPad applications [29–44], three digital cameras; [45–47] whilst two studies used a combination of visual acuity charts and questionnaires [48, 49]. The included studies reported different forms of amblyogenic conditions including high refractive errors, astigmatism, ocular misalignment, and leukocoria. For these conditions except leukocoria, there are different criteria used as a cut-off point for amblyopia detection. Age of patients included in studies ranged from 0 to 16 years old.

All 28 studies included a reference standard except one longitudinal study, which compared facial photographs of patients with and without leukocoria. 22 studies had full ophthalmological examination whilst 6 studies had validated visual acuity measurement tools as their reference standard.

Of the 27 studies included, the sample size ranged from 70 to 36,973 patients, with a mean of 2004 patients. A summary of the studies included in the systematic review is presented in Table 1.

**Table 1:** Summary of studies grouped by type of home-based screening tool.

Author	Name of diagnostic test	Country of Origin	Type of Study	Age groups	Number of patients	Gold standard	Sensitivity	Specificity	Positive likelihood ratio	Negative likelihood ratio
**Internet-based tests**										
Briscoe et al. [22]	Computer-based	Israel	Prospective study	4–6 years old	294	Standard visual acuity (symbol) and stereopsis test	Visual acuity tests (symbol): 81.5% Stereopsis: 50%	Visual acuity tests (symbol): 83% Stereopsis: 98.9%	Visual acuity tests (symbol): 4.794 Stereopsis: 45.455	Visual acuity tests (symbol): 0.223 Stereopsis: 0.506
Longmuir et al. [23]	Visionforkids.org	United States of America	Prospective, randomised study	3–12 years old	203	Validated electronic visual acuity protocol	78.7% (95% CI: 66–87.7)	89.4% (95% CI: 82.9–93.8)	7.2	0.24
Schlenker et al. [24]	LazyeyeTest.org	Canada	Prospective, masked cross over study	4–5 years old	70	Full ophthalmological examination	88%	92%	11	0.13
Thomson et al. [25]	Questionnaire and Computer system algorithm	United Kingdom	Prospective study	5–8 years old	245	Full ophthalmological examination	93.8%	96.1%	24.05	0.065
Trivedi et al. [26]	EyeSpy (video game) and Electronic Visual Acuity tester	United States of America	Prospective study	8–16 years old	72	Full ophthalmological examination	EyeSpy (with patch) compared with professional examination: 88%(68–97) EyeSpy (with goggles) compared with professional examination: 88% (68–97) EVA/stereopsis compared with professional examination: 88% (68–97)	EyeSpy (with patch) compared with professional examination: 87% (73–95) EyeSpy (with goggles) compared with professional examination: 74% (59–85) EVA/stereopsis compared with professional examination: 94% (81–98)	EyeSpy (with patch) compared with professional examination: 6.8 (3.21–14.75) EyeSpy (with goggles) compared with professional examination: 3.4 (2.07–5.73) EVA/stereopsis compared with professional examination: 13.8 (4.57–41.6)	EyeSpy (with patch) compared with professional examination: 0.13 (0.04–0.40) EyeSpy (with goggles) compared with professional examination: 0.16 (0.05–0.47) EVA/stereopsis compared with professional examination: 0.13 (0.04–0.37)
Yamada et al. [27]	Jaeb Visual Acuity Screener (JVAS)	United States of America	Prospective study	3 to 7 years old	186	Full ophthalmological examination	88–93%	70–86%	2.9	0.4
Qin et al. [28]	Autoacuity tester	China	Prospective study	3 to 13 years old	552	Tumbling E ETDRS for school children, Lea Symbols and TAC for pre-school children.	88 %(95% CI 75–95)	92% (95% CI 80–97)	11	0.13
**Mobile applications**										
Arnold et al. 2018 [29]*	Gobiquity app/GoCheckKids on Nokia model 1020 smartphones.	United States of America	Prospective study	5 to 8 years old	6310	Full ophthalmological examination	–	–	–	–
Arnold et al. 2014 [30]	GoCheckKids (Gobiquity, Aliso Viejo, CA) for the iPhone 4s (Apple,Cupertino, CA)	United States of America	Prospective study	9–146 months old	108	Full ophthalmological examination	81%	91%	9	0.21
Arnold et al. 2018 [31]	GoCheck Kids (GCK, Gobiquity Mobile Health, Scottsdale, AZ, USA)	United States of America	Prospective, multicentre study	1–6 years old	287	Full ophthalmological examination	Manual grading: 76% (95% CI 71–81%) Automatic grading: 65% (95% CI 62–68%)	Manual grading: 85% (95% CI 80–90%) Automatic grading: 83% (95% CI 80–86%)	Manual grading: 5.067 Automatic grading: 3.824	Manual grading: 0.282 Automatic grading: 0.422
Cheng et al. [32]	EyeTurn on iphone7	China	Prospective, cross-sectional study	6–10 years old	133	Full ophthalmological examination–not all patients underwent this	With detection of strabismus of 3PD: Best sensitivity- 83%	With detection of strabismus of 3PD: Best specificity- 76.5%	3.55	0.22
Foggia et al. [33]	Vision test on touchpad developed by Strasbourg University	France	Prospective monocentric concordance study	3–10 years old	101	Standard visual acuity test (Monoyer, the Snellen-E, Pigassou) at 5m	92%	80%	4.5	0.1
Gupta et al. [34]	Smartphone (OPPOA37f) with camera specification of 8MP	India	Prospective, cross-sectional study	5–8 years old	250	Full ophthalmological examination	93.2–94.9%	90.5–91.6%	10.33	0.077
Law et al. [35]*	GoCheck Kids on Nokia Lumia 1020 phone (Nokia Corporation, Espoo, Finland)	United States of America	Retrospective study	6 months to 6 years old	2963	Full ophthalmological examination-not all patients underwent this	–	–	–	–
Levitt et al. [36]	GoCheck Kids iPhone 7+ smartphone with flash concentrating case with nonaccommodative glow fixation (GCK glow)	United States of America	Prospective study	Less than 8 years old	131	Full ophthalmological examination	78%	63%	2.11	0.35
Martin et al. [37]	GoCheck Kids	United States of America And Myanmar	Reliability Analysis	Median age 8.2 years	162	Visual acuity with HOTV and cycloplegic refraction	2013 ARF criteria: 63% including inconclusive referrals	2013 ARF criteria: 83% including inconclusive referrals	3.71	0.45
Mesquita et al. [38]	Mhealth application	Brazil	Concordance study	5–15 years old	224	Examination by strabismus ophthalmologist	Considering cutoff point of 6PD: 89.47% (95% CI = [66.8%; 98.7%]) Considering cutoff point of 11PD: 68.75% (95% CI = [41.3%; 88.9%])	Considering cutoff point of 6PD: 84.39% (95% CI = [78.68%; 89%]) Considering cutoff point of 11PD: 93.27% (95% CI = [88.96%; 96.27%])	Considering cutoff point of 6PD: 5.732 Considering cutoff point of 11PD: 10.215	Considering cutoff point of 6PD: 0.125 Considering cutoff point of 11PD: 0.335
^,^Munson et al^$^ [39]	CRADLE app on iphoneX	United States of America	Retrospective longitudinal study	Less than 1 month– 2 years old	40 children or 52,982 facial photo-graphs	Not applicable	90.0% (95% CI 76.9–100%) for below 2 years old	20% (95% CI 2.5–37.5%) for below 2 years old	1.12	0.5
Nik Azis et al. [40]	AAPOS Vision Screening app (Lea Symbols)	Malaysia	Prospective cross-sectional study	5–6 years old	200	Lea symbols on standard charts Full examination for VA worse than logmar 0.26	79.5% (95% confidence interval [CI] 68.8–87.8%)	71.8% (95% CI 62.7–79.7%)	2.86	0.28
Peterseim et al. [41]	GoCheck preloaded Nokia Lumia 1020, software version 4.6 with image processing version R4d.	United States of America	Prospective study	6 months-6 years old	206 children	Full ophthalmological examination	76.0% (95% CI 64.6–85.1%)	67.2% (95% CI 58.4–75.1%)	2.3	0.49
Vagge et al. [42]	CRADLE app on iphone 7	Italy	Prospective study	2–6 months	244 eyes from 122 children	Full ophthalmological examination	15.38% (CI: 1.92–45.45)	100% (CI 98.48–100)	∞	0.85
Venecia et al. [43]	Peek acuity	United States of America	Prospective study	6–16 years	393	Full ophthalmological examination	47%	83%	2.77	0.64
Walker et al. [44]	GoCheck Kids on iphone 2018	United States of America	Prospective study	6 months –6 years old	244	Full ophthalmological examination	90.5% (95% CI:81.5–96.1) for only gradable images	68.1% (95% CI: 60.3–75.3)	2.84	0.13
**Digital camera**										
Arnold et al. 2004 [45]	DV-S20 digital camera	United States of America	Prospective study	6 months-10 years old	622	Full ophthalmological examination	91%	98%	45.5	0.092
Granet et al. [46]	Modified digital camera	United States of America	Prospective, cross-sectional study	9 months to 16 years old	206	Full ophthalmological examination	89%	82.9%	5.21	0.13
Guo et al. [47]	50 mm catadioptric lens mounted on DCS 410 Kodak digital camera connected to PC computer	China	Prospective, study	9–50 months old	300	Full ophthalmological examination	94.6%	90.1%	9.5	0.056
**Others**										
Lan et al. [48]	A combination of near visual acuity chart and questionnaires	China	Prospective, randomised study	3–6 years old	2442	Full ophthalmological examination	80% (95% CI: 59.8–100)	94.1% (95% CI: 93.8–94.4)	13.33	0.21
Lim et al. [49]	Picture optotype chart and questionnaire	South Korea	Prospective study	3–5 years	36 973	Full ophthalmological examination	45%	92.5%	6.43	0.59

*no sensitivity, specificity values were published.

^$^This is a retrospective longitudinal study therefore no gold standard test was used, excluded from QUADAS-2 risk of bias assessment.

Sensitivity and specificity values were reported in 25 studies. Confusion matrices were included in 16 studies and can be assessed through Online Supplementary Table 2.

### Risk of bias assessment

We used the QUADAS-2 tool in the systematic appraisal of all but one longitudinal study which did not include a reference standard or index test (Table 2) [39].

**Table 2:** Risk of bias assessment using QUADAS-2 tool [22–49].

With regards to patient selection, 52% of included studies were shown to have high applicability concern. This was primarily due to patient selection from tertiary centres which were not representative of the normal population. A similar proportion showed unclear or high risk of bias in patient selection as the process was either unclear or non-systematic. Around a quarter of studies showed unclear or high risk of bias and applicability concern in index test used: 18.5% showed unclear or high risk of applicability concern in reference test used, whilst 33.3% showed unclear or high risk of bias in reference test used.

15% of studies were also shown to have a high risk of bias in flow and timing, as not all patients who went through the index test were subjected to the reference test or were lost to follow up.

### Quantitative synthesis

Specificity and sensitivity varied across studies with regards to the type of diagnostic tests used, country of study, patient selection and age of children included. Sensitivity estimates for internet-based tests ranged from 50.0% [22] to 93.8% [25] while specificity estimates ranged from 70.0% [27] to 98.9% [22]. Sensitivity estimates for mobile applications were between 15.4% [42] to 94.9% [34], while specificity estimates were between 63.0% [36] to 93.0% [38]. Digital camera as index test showed sensitivity estimates between 89.0% [46] to 94.6% [47] and specificity estimates between 82.9% [46] to 98% [45].

**Fig. 2:**
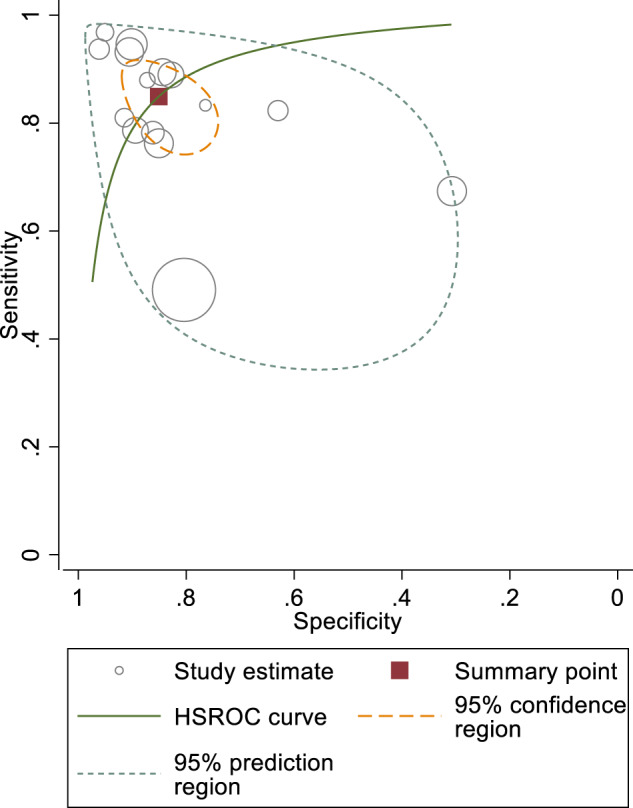
Summary of sensitivity and specificity estimates through a HSROC curve. A line of best fit, which is the HSROC curve, passes through these dots. The size of dots represents the size of study included. The region marked by the dotted lines represents the 95% confidence interval for the studies included.

A meta-analysis could not be carried out as the studies were heterogenous in terms of type of screening tool used, differing cut-off values for amblyogenic conditions detected, population, reference standard used, and lack of raw data from confusion matrices. The heterogeneity of studies included is demonstrated in Fig. 2.

## Discussion

### Summary

To the best of our knowledge, this represents the first systematic review on the diagnostic accuracy of home-based amblyopia screening tools. Our systematic review revealed that there is a broad variety of home-based amblyopia screening tools available globally in the form of internet-based tests, mobile applications, digital cameras, and a combination of others. However, based on our rigorous assessment on the quality of studies included, there is an overall risk of bias in the existing studies. Lack of raw data pertinent for a pooled analysis, heterogeneity in the studies, and lack of standardisation on age-defining values for detecting amblyogenic conditions makes meaningful comparison of sensitivity and specificity estimates limited, which is crucial for the systematic appraisal of diagnostic accuracy studies [50].

### Quality of evidence

Patient selection in around half of the studies demonstrated unclear or high risk of bias, either due to patient selection from tertiary centres, or non-systematic methods of recruitment. The paediatric clinic represents a population that could have a higher prevalence of amblyogenic risk factors. This may lead to overestimation of sensitivity values. Less than half of the studies showed unclear or high risk of bias in the index and reference test used. This is due to the lack of clarity on blinding during the conduct of the index test, or its interpretation which is not independent of the reference test, and the lack of uniformity in the reference test used across the studies. In studies that included full ophthalmological examination as their reference test, most included slit lamp and fundus examination by ophthalmologists, but not all specified whether retinoscopy, cover tests, or assessment of ocular motility were included. It was also difficult to standardise the visual acuity measurement tools used for studies that selected this as their reference standard, as the modality of tests would depend on the age of children recruited. In addition, there is lack of standardisation and international consensus on the age-defining values for detecting amblyogenic conditions such as high refractive errors, astigmatism, and ocular misalignment. Response bias was also present in studies that used a combination of screening tools, including questionnaires, as this depended on the response rate of the population involved [25, 48, 49].

### Internet-based tests

Five studies published computer algorithm-generated standard visual acuity tests [22–25, 28], one study involved the use of video game (Eyespy) [26], and another study used computer generated checkboards of various frequencies for amblyopia detection [28]. Among these, only Longmuir et al. [23], Schlenker et al. [24], and Trivedi et al. [26] included lay screeners or parents in the conduct of the index test. The Jaeb Visual Acuity Screener (JVAS) [27], a free internet-based visual acuity screening test has been developed and validated for use among non-ophthalmic health professionals by the PEDIG group.

### Mobile applications

The most common mobile application from the diagnostic accuracy studies included was GoCheck Kids (Gobiquity Mobile Health, Scottsdale, AZ, USA) [29–31, 35–38, 41, 44]. One of the advantages of mobile applications for screening is it can be used for younger children, such as the CRADLE app [39, 42] which is used on children as young as two months old to detect leukocoria. However, it demonstrated low sensitivity as it does not detect other amblyogenic risk factors with higher prevalence. In contrast, the GoCheck Kids app and Peek acuity app were used to detect high refractive errors and astigmatism, which were more common in the study population, hence reporting higher sensitivity values. The Eyeturn app [32] and Mhealth [38] were both used to detect ocular misalignment. Table 3 summarises the existing internet-based tests or mobile applications from our systematic review which has gone through validation studies and are free of charge.

**Table 3:** Validated tablet or mobile home screening tools.

	VA Method	Testing Distance	Device	Validation studies	Limitations
**GoCheck Kids**	HOTV ETDRS Crowded	5 feet	iOS Android	Silverstein et al. [56]	The lowest acuity that can be measured at 5 feet is 20/63 (0.5 logMAR). It also requires a special adaptation for the camera, and as the phone is supplied by the company, this limits its availability
**Peek Acuity**	Tumbling E Boxed	2 meters	Android	Venecia et al. [43] Bastawrous, H.K et al. [57] Zhao et al. [58] Rono et al. [51]	Younger children may not be able to appreciate and communicate the orientation of the tumbling E letters
**Jaeb Visual Acuity Screener**	HOTV ETDRS Crowded	5 feet	Windows	Yamada et al. [27]	Requires internet connectivity, windows on computer or tablet

Peek Acuity is the only app to date which had been shown through a cluster randomized controlled trial to increase follow-up rates through an integrated system involving the app [51].

### Digital camera

There were only three studies that used digital camera for amblyopia screening. One incorporated a DV-S20 [45], and the other two utilised modified digital camera [46, 47]. However, the interpretation of the images was either by professionals or based on computer-generated analysis, and they tend to be more expensive than internet or mobile applications, making them less ideal as home-based screening tools.

### Research in context

This systematic review is the first to publish a descriptive summary on the diagnostic accuracy of the latest available home-based amblyopia screening tools. There are parallels on the use of telemedicine rapidly in the COVID-19 era for the diagnosis and management of diabetic retinopathy, glaucoma, age-related macular degeneration, and for triage in emergency eye care [52]. Amblyopia detection to date has been largely undertaken by trained ophthalmic professionals. Even though the advent of photoscreeners such as the Plusoptix™, SPOT™, and autorefractors have enabled the screening process to be easier and less time-consuming, there is a lack of strong evidence on its cost-effectiveness for use in the community [53]. Home-based tools for amblyopia screening satisfies the WHO criteria for a good screening test [54]. Amblyopia has a pre-clinical phase that enables early detection and is a condition that can be treated. Home-based screening tools are easily available, less costly, or free of change, and reduce the dependence on trained ophthalmic professionals. Our systematic review showed that the sensitivity and specificity of some home-based tools to be comparable to photoscreeners or autorefractors [55]. As there has been a lot of research into the effectiveness of home-based tools for detection of amblyopia, this systematic review is important to summarise the existing evidence from literature in this field.

### Strengths and limitations

This systematic review has highlighted key findings which may help pave the way for further research using home-based tools in amblyopia screening. The home-based tools included in this review are not restricted to the digital age, as questionnaires, digital cameras, and visual acuity charts used in the home setting are also included. Even though this systematic review included a comprehensive range of home-based tools in diagnostic accuracy studies, we did not include any studies reporting validity or reliability of home-based amblyopia screening tools or studies evaluating the cost-effectiveness of these tools. In addition, there is a possibility that other validated home-based tools are not captured in this systematic review if they have not been evaluated in diagnostic accuracy studies. Moreover, studies examining the feasibility of these tools by lay screeners were not included. Some of these internet-based tools or mobile applications may require some training before use, which may exclude some users.

## Conclusions

This systematic review highlighted the availability of home-based screening tools, which could aid in amblyopia screening. However, there is a need to improve the quality and reporting of diagnostic accuracy studies using these tools. Home-based screening tools could be advantageous especially due to the COVID-19 pandemic, where amblyopia screening has stalled. Such tools may be a suitable option for low- and middle-income countries. However, as the incentive is upon the parents or lay screeners to utilise these resources, there is a need to educate the public on the importance of amblyopia screening at home, given the various options available for this. Ideally, evidence-based amblyopia screening tools could made be widely available for home-use, but further work is needed to identify the most effective tools for this purpose.

## Supplementary information

online supplementary table 1

online supplementary table 2

online supplementary appendices 1-4
