# Supplementary material for: Home-based screening tools for amblyopia: a systematic review
Source: Eye (Lond). 2023 Feb 24;37(13):2649–58. doi: 10.1038/s41433-023-02412-3 (PMC9951845; doi:10.1038/s41433-023-02412-3)
Supplement: Supplementary file 1 — online supplementary table 1 [file 41433_2023_2412_MOESM1_ESM.docx]

**Studies excluded after screening full text**

A=Wrong population

B=Wrong intervention/study

C=Wrong outcome measures

D= Conference abstract/pre-print only

E= Tool does not qualify as home-based screening tool

F= No results available

| **No** | **Source** | **Author** | **Title** | **Reason** |
| --- | --- | --- | --- | --- |
| 1. | Medline | Van Eenwyk et al | Artificial intelligence techniques for automatic screening of amblyopic risk factors | E |
| 2. | Medline | Tittes et al | Assessment of stereovision with digital testing in adults and children with normal and impaired binocularity | C |
| 3. | Medline | Stewart et al | Comparison of logMAR ETDRS chart and a new computerised staircased procedure for assessment of the visual acuity of children | B Validation study |
| 4. | Medline | Moke et al | Computerized method of Visual Acuity Testing: Adaptation of the Amblyopia Treatment Study Visual Acuity Testing Protocol | B Validation study |
| 5 | Medline | Matsuo et al | Detection of Strabismus and Amblyopia in 1.5- and 3-year-old Children by a Preschool Vision-screening Program in Japan | B |
| 6 | Medline | Kennedy et al | Evaluation of the iscreen digital screening system for amblyopic factors | E |
| 7 | Medline | Hay et al | Retinal Reflex Photometry as a Screening Device for Amblyopia and Preamblyopic States in Children | E |
| 8 | Medline | Paysse et al | Parent-Administered Visual Acuity Testing: Is it Reliable and Can it Improve Office Efficiency? | B Validation study |
| 9 | Medline | Maslin et al | Photoscreening to detect amblyopia | E |
| 10 | Medline | Wu et al | Portable acuity screening for any school: validation of patched HOTV with amblyopic patients and Bangerter normals | B Validation study |
| 11 | Medline | Gupta et al | Smartphone photography for screening amblyogenic conditions in children | E |
| 12 | Medline | Silverstein et al | Teleophthalmology: Evaluation of Phone-based Visual Acuity in a Pediatric Population | B Validation study |
| 13 | Medline | Tonner et al | The Handy Eye Check: a mobile medical application to test visual acuity in children | B Validation study |
| 14 | Medline | Taylor et al | User-Centered Development of a Web-Based Preschool Vision Screening Tool | B |
| 15 | Medline | Laidlaw et al | Validation of a computerised logMAR visual acuity measurement system (COMPlog): comparison with ETDRS and the electronic ETDRS testing algorithm in adults and amblyopic children | B Validation study |
| 16 | Medline | Bregman et al | Validation of photoscreening technology in the paediatrics office | E |
| 17 | Medline | Bani et al | Beyond photography: Evaluation  of the consumer digital camera to  identify strabismus and anisometropia  by analyzing the Bruckner’s reflex | A |
| 18 | Embase | Horwood et al | Costs and effectiveness of two models of school- entry visual acuity screening in the UK | B |
| 19 | Embase | Dahlmann-Noor et al | Real-time automatic strabismus screening using digital image analysis techniques | B |
| 20 | Embase | Struble Jr et al | Efficacy of a vision-screening tool for birth to 3 years early intervention programs | B |
| 21 | Embase | Silbert et al | Use of digital photoscreeners to detect amblyopia risk factors(ARFs): a multidisciplinary evidence-based approach—care and cost implications. | D (conference abstract) |
| 22 | Embase | Silbert et al | Do we need to directly detect astigmatism when photoscreening for amblyopic risk factors (ARF)? | D (conference abstract) |
| 23 | Embase | Chen et al | Pilot testing of a multi-functional device for pediatric vision screening application | D (conference abstract) |
| 24 | Embase | Onoe et al | Instrument-based visual screening of 3-year old children in Japan | D (conference abstract) |
| 25 | Embase | Gusek et al | Sensitivity of assessment of refraction with infrared-videorefractor VRB 200. [German] | C |
| 26 | Embase | Zhang et al | Application of the digital photo refraction in the screening of amblyopiogenic risk factor for preschool children. | D (conference abstract) |
| 27 | Embase | Aldossary et al | Preschool vision screening by parents using an iPad in Saudi Arabia | D (conference abstract) |
| 28 | Embase | Arnold et al | iCheckKids, SPOT, iScreen, and Plusoptix performance in a high-risk, young pediatric eye practice | D (conference abstract) |
| 29 | Embase | Arnold et al | Efficacy of a mobile smart phone vision-screening device with automated image-processing analysis in the evaluation of amblyopia risk factors in preschool children | D (conference abstract) |
| 30 | Embase | Butler et al | Evaluation of a novel digital infant acuity test | D(conference abstract) |
| 31 | Embase | Friendly et al | An Automated Visual Acuity Testing Computer Program Using the Apple II System | B |
| 32 | Embase | Kane et al | Automated brightness sense screening for amblyopia | D (conference abstract) |
| 33 | Embase | Kane et al | Automated vision screening of children using a mobile graphic device | D (pre-print) |
| 34 | Embase | Pimentel et al | Cycloplegic refractive error and amblyopia risk factors found after photo-screening. | D(conference abstract) |
| 35 | Embase | Yamada et al | Comparing Failure Criteria for a New PC-Based Pediatric Vision-Screening Test | D (conference abstract) |
| 36 | Embase | Park et al | Validation of iPad-based simplified visual acuity testing in children | D(conference abstract) |
| 37 | Embase | Shah et al | Validity of a layperson-administered Web-based vision screening test for the detection of amblyopia in the home environment | D(conference abstract) |
| 38 | Embase | Vaughan et al | Photoscreening for refractive error and strabismus with a smartphone app | D (conference abstract) |
| 39 | Embase | Srinivasan et al | Analysis of a novel method for detection of vision disorders in children birth to three years of age | D (conference abstract) |
| 40 | Embase | Aslam et al | Automated Measurement of Visual Acuity in Pediatric Ophthalmic Patients Using Principles of Game Design and Tablet Computers | B Validation study |
| 41 | Embase | Toner et al | The Handy Eye Check: a mobile medical application to test visual acuity in children | B Validation study |
| 42 | Embase | Zhao et al | Smartphone app correlates well with standard visual screening assessment | D (short article) |
| 43 | Embase | Gorham et al | Comparison of two photo-screeners in a population of Syrian  refugee children. | D (conference abstract) |
| 44 | Web of Science | Braun et al | Visual Function Testing and Training for Children with Different Visual Impairments, by Using a Software Interface, Serious Game Type for Laptop and Tablet | C |
| 45 | Web of Science | Weise et al | Evaluation of computer-based testing for aniseikonia in children | A |
| 46 | Web of Science | Racano et al | Validation of the 2WIN corneal reflexes app in children | B |
| 47 | Web of Science | Khumdat et al | Development of a computer system for strabismus screening | B |
| 48 | Web of Science | Arnold et al | Calibration and validation of nine objective vision screeners with contact-lens induced anisometropia | B |
| 49 | Web of Science | Agarwala et al | Utilizing minicomputer technology for low-cost photorefraction: a feasibility study | B |
| 50 | Web of Science | Wright et al | Is community screening for amblyopia possible, or appropriate? | C |
| 51 | Web of Science | Kupl et al | The Electronic Visual Acuity Tester: Testability in Preschool Children | B Validation study |
| 52 | Web of Science | Ma et al | A One-Step, Streamlined Children’s Vision Screening Solution Based on Smartphone Imaging for Resource-Limited Areas: Design and Preliminary Field Evaluation | B |
| 53 | Web of Science | Ma et al | Reliability and Validity of an Automated Computerized Visual Acuity and Stereoacuity Test in Children Using an Interactive Video Game | B |
| 54 | Web of Science | Rodriguez-Vallejo et al | Visual acuity and contrast sensitivity screening with a new iPad application | A |
| 55 | Web of Science | Van Eenwyk J et al | Automated human vision assessment using computer vision and artificial intelligence | E |
| 56 | Web of Science | Chun J et al | Deep learning–based prediction of refractive error using photorefraction images captured by a smartphone: model development and validation study | E |
| 57 | Pubmed | Pueyo et al | Development of a system based on artificial intelligence to identify visual problems in children: study protocol of  the TrackAI project | B |
| 58 | Pubmed | Painter et al | Parental Home Vision Testing of Children During the COVID-19 Pandemic | B Validation study |
| 59 | Pubmed | Ritchie et al | Can Visual Acuity Be Reliably Measured at Home? Validation of Telemedicine Remote Computerised Visual Acuity Measurements | B Validation study |
| 60 | Pubmed | O’Connor et al | Evaluation of a New Method to Track Changes in Vision at Home for Children Undergoing Amblyopia Treatment | B Validation study |
| 61 | Pubmed | Chen et al | Eye-tracking-aided digital system for strabismus diagnosis | B |
| 62 | Pubmed | Wisse et al | Validation of an Independent Web-Based Tool for Measuring Visual Acuity and Refractive Error (the Manifest versus Online Refractive Evaluation Trial): Prospective Open-Label Noninferiority Clinical Trial | A |
| 63 | Pubmed | Murali et al | Effectiveness of Kanna photoscreener in detecting amblyopia risk factors | E |
| 64 | Pubmed | Murali et al | Application of deep learning and image processing analysis of photographs for amblyopia screening | E |
| 65 | Cochrane | Solaka, N et al | Comparison of a new prototype of netra-g cell phone-based refraction with subjective refraction | B |
| 66 | Clinical Trials.gov | PI: Sean Donahue | Evaluation of a Smartphone Application, GoCheckKids™ as a Photo Screening Tool in a Pediatric Population | F |
| 67 | Clinical Trials.gov | Sponsor:  Alaska Blind Child Discovery | Comparative Validation of "Blinq" and "2WIN" Vision Screeners | E |
| 68 | Clinical Trials.gov | Principal Investigator:Martha A Howard | Gocheck Kids vs. Welch Allyn Spot Vision Screener | F |
| 69 | Clinical Trials.gov | Principal Investigator:Antoine Sylvestre-Bouchard | Performance of a Photoscreener for Vision Screening in a Haitian Pediatric Population | E |
