# Supplementary material for: Home-based screening tools for amblyopia: a systematic review
Source: Eye (Lond). 2023 Feb 24;37(13):2649–58. doi: 10.1038/s41433-023-02412-3 (PMC9951845; doi:10.1038/s41433-023-02412-3)
Supplement: Supplementary file 3 — online supplementary appendices 1-4 [file 41433_2023_2412_MOESM3_ESM.docx]

# **Appendices**

## **Appendix 1:**

## **Search strategy**

## **a) MEDLINE**

1. exp Amblyopia/ or amblyop*.mp. or amblyog*.mp.

2. ((assess* or diagnos* or detect* or screen* or test*) adj4 (vision* or visual*)).mp.

3. exp visual acuity/

4. exp vision screening/ or screen*.mp.

5. ((assess* or diagnos* or detect*or screen* or test*) adj4 (communit* or population*)).mp.

6. ((assess* or diagnos* or detect* or screen* or test*) adj4 program*).mp.

7. 2 or 3 or 4 or 5 or 6

8. (home* or internet* or web* or app* or computer* or smartphone* or mobile*).mp. or Mobile Applications/ or Smartphone Applications/

9. 1 and 7 and 8

10. limit 9 to "all child (0 to 18 years)"

**b) Embase**

1. exp Amblyopia/ or amblyop*.mp. or amblyog*.mp.

2. ((assess* or diagnos* or screen* or test*) adj4 (vision* or visual*)).mp.

3. exp vision test/

4. exp vision screening/ or screen*.mp.

5. ((assess* or diagnos* or screen* or test*) adj4 (communit* or population*)).mp.

6. ((assess* or diagnos* or screen* or test*) adj4 program*).mp.

7. 2 or 3 or 4 or 5 or 6

8. (home* or internet* or web* or app* or computer* or smartphone* or mobile*).mp. or Mobile Applications/ or Smartphone Applications/

9. (infan* or child* or toddler* or paediat* or pediat* or infant* or newborn* or preschool* or neonat*).mp.

10. 1 and 7 and 8 and 9

**c) Cochrane search strategy**

Search

#1 MeSH descriptor: [Amblyopia] explode all trees

#2 amblyop* or amblyog*

#3 #1 or #2

#4 MeSH descriptor: [Vision Tests] explode all trees

#5 MeSH descriptor: [Vision Screening] explode all trees

#6 (assess* or diagnos* or screen* or test*) near/4 (vision* or visual*) or screen*

#7 (assess* or diagnos* or screen* or test*) near/4 (communit* or population*)

#8 (assess* or diagnos* or screen* or test*) near/4 (program*)

#9 #4 or #5 or #6 or #7 or #8

#10 MeSH descriptor: [Mobile Applications] explode all trees

#11 home* or internet* or web* or app* or computer* or smartphone* or mobile*

#12 #14 or #15

#13 #3 and #9 and #12

#14 infan* or child* or toddler* or paediat* or pediat* or infant* or newborn* or preschool* or neonat*

#15 #13 and #14

## **d)Web of Science Core Collection**

(amblyop* or amblyog*) *AND* **TOPIC:** ("visual acuity" or " vision screening" or vision* or visual* or screen*) *AND* **TOPIC:** (home* or internet* or web* or app* or computer* or smartphone* or mobile* or " mobile application*" or " smartphone application*") *AND* **TOPIC:** (infan* or child* or toddler* or paediat* or pediat* or infant* or newborn* or preschool* or neonat*)

**Timespan:** All years. **Indexes:** SCI-EXPANDED, SSCI, A&HCI, CPCI-S, CPCI-SSH, BKCI-S, BKCI-SSH, ESCI, CCR-EXPANDED, IC.

## **e)PubMed**

("amblyopia"[MeSH Terms] OR "amblyo*"[All Fields]) AND ("visual acuity"[All Fields] OR "vision screening"[All Fields] OR "vision*"[All Fields] OR "visual*"[All Fields] OR "screen*"[All Fields]) AND ("home*"[All Fields] OR "internet*"[All Fields] OR "web"[All Fields] OR "app"[All Fields] OR "computer*"[All Fields] OR "smartphone*"[All Fields] OR "mobile*"[All Fields] OR "mobile application*"[All Fields] OR "smartphone application*"[All Fields]) AND ("infan*"[All Fields] OR "child*"[All Fields] OR "toddler*"[All Fields] OR "paediat*"[All Fields] OR "pediat*"[All Fields] OR "newborn*"[All Fields] OR "preschool*"[All Fields] OR "neonat*"[All Fields])

## **f)Clinicaltrials.gov**

(Amblyop* or amblyog*) AND ("vision test" or " vision screening" or vision* or visual*or screen*) *AND* ( home* or internet* or web* or app* or computer* or smartphone* or mobile* or " mobile application*" or " smartphone application*") limit to child birth to 17

## **Appendix 2: Screening questionnaire**

**Instructions for screeners:** Tick the appropriate box per screening question. If “no” at any stage, exclude. If “yes” or “unclear”, proceed to next stage; if “yes” at Stage 3, include. If “unclear” at Stage 3, contact study authors for further information and/or seek verdict from third arbitrator.

**Stage 1: Title Screening**

1a) Does the study represent Level IV evidence or above, i.e. case series, cohort studies, case-control studies, randomised controlled trials (RCTs) and systematic reviews?

| Yes |  |
| --- | --- |
| No |  |
| Unclear |  |

1b) Does the study involve home-based screening methods for amblyopia in children under 18 years of age?

| Yes |  |
| --- | --- |
| No |  |
| Unclear |  |

**Stage 2: Abstract Screening**

2a) Does the study represent Level IV evidence or above, i.e. case series, cohort studies, case-control studies, randomised controlled trials (RCTs) and systematic reviews?

| Yes |  |
| --- | --- |
| No |  |
| Unclear |  |

2b) Does the study involve home-based screening methods for amblyopia in children under 18 years of age?

| Yes |  |
| --- | --- |
| No |  |
| Unclear |  |

**Stage 3: Full text Screening**

3a) Does the study represent Level IV evidence or above, i.e. case series, cohort studies, case-control studies, randomised controlled trials (RCTs) and systematic reviews?

| Yes |  |
| --- | --- |
| No |  |
| Unclear |  |

3b) Does the study involve home-based screening methods for amblyopia in children under 18 years of age?

| Yes |  |
| --- | --- |
| No |  |
| Unclear |  |

## **Appendix 3: Data extraction tool, adapted from the Cochrane Collaboration**

Methods

| Aim of study |  |
| --- | --- |
| Study design |  |
| Inclusion criteria |  |
| Exclusion criteria |  |
| Methods of recruitment |  |
| Methods of randomisation (if applicable) |  |
| Number of patients |  |

Specific to diagnostic accuracy studies

| Personnel conducting index test |  |
| --- | --- |
| Personnel conducting gold standard test |  |
| Subjects receiving test |  |
| Blinding (if applicable) |  |
| Index test |  |
| Reference test |  |
| Personnel interpreting test results |  |
| Withdrawal rate/loss to follow up  If not specified state so |  |
| Sensitivity(including CI) |  |
| Specificity(including CI) |  |
| False positive |  |
| False negative |  |
| Correlation coefficient including p-values |  |

Specific to other evaluation studies

| Personnel conducting index test |  |
| --- | --- |
| Personnel conducting reference test |  |
| Subjects receiving test |  |
| Blinding (if applicable) |  |
| Index test |  |
| Reference test |  |
| Personnel interpreting test results |  |
| Withdrawal rate/loss to follow up |  |
| Results reported (appropriate statistical measures ) |  |
| Economic consideration/cost required |  |

## **Appendix 4: QUADAS-2 tool**

Domain 1: Patient selection

| A. Risk of bias  Describe methods of patient selection: | Low | High | Unclear |
| --- | --- | --- | --- |
| Was a consecutive or random sample of patients enrolled? |  |  |  |
| Was a case-control design avoided? |  |  |  |
| Did the study avoid inappropriate exclusions? |  |  |  |
| Could the selection of patients have introduced bias? |  |  |  |
| B. Concerns regarding applicability | Low | High | Unclear |
| Is there concern that the included patients do not represent the actual spectrum of patients in practice? |  |  |  |

Domain 2: Index test(s)

| Risk of bias  Describe the index text and how is it conducted and interpreted: | Low | High | Unclear |
| --- | --- | --- | --- |
| Were the index test results interpreted without knowledge of the results of the reference standard? |  |  |  |
| If a threshold was used, was it pre-specified? |  |  |  |
| Was the index test described in sufficient detail to enable replication of the test? |  |  |  |
| Could the index test, its conduct, or its interpretation have introduced bias? |  |  |  |
| B. Concerns regarding applicability | Low | High | Unclear |
| Is there concern that the index test, its conduct, or interpretation differ from the review question? |  |  |  |

Domain 3: Reference standard

| Risk of bias  Describe the index text and how is it conducted and interpreted: | Low | High | Unclear |
| --- | --- | --- | --- |
| Is the reference standard likely to correctly classify the target condition? |  |  |  |
| Were the reference standard results interpreted without knowledge of the results of the index test? |  |  |  |
| Was the standard test described in sufficient detail to enable replication of the test? |  |  |  |
| Could the reference standard, its conduct, or its interpretation have introduced bias? |  |  |  |
| B. Concerns regarding applicability | Low | High | Unclear |
| Is there concern that the target condition as defined by the reference standard does not match the review question? |  |  |  |

Domain 4: Flow and timing

| Risk of bias | Low | High | Unclear |
| --- | --- | --- | --- |
| Was there an appropriate interval between index test(s) and reference standard, so that the target condition did not change between two tests? |  |  |  |
| Did all patients receive a reference standard? |  |  |  |
| Did all patients receive the same reference standard? |  |  |  |
| Were all patients included in the analysis? |  |  |  |
| Could the patient flow have introduced bias? |  |  |  |
